# Disseminated meningococcal infection presenting with constrictive pericarditis and bilateral septic arthritis without meningitis: A case report

**DOI:** 10.1177/2050313X261488253

**Published:** 2026-09-06

**Authors:** Mutasim Binidris, Menatalla Shehata, Ahmed Mahfouz, Omar Abousaad, Willington Francis, Muhammed Rafeeq

**Affiliations:** 1 Department of Medical Education, 36977 Hamad Medical Corporation, Doha, Qatar; 2 Department of Clinical Pharmacy, Heart Hospital, 36977 Hamad Medical Corporation, Doha, Qatar; 3 College of Pharmacy, 61780 Qatar University, Doha, Qatar; 4 Department of Genomics and Precision Medicine, 370593 Hamad Bin Khalifa University, Doha, Qatar; 5 Department of Radiology, 36977 Hamad Medical Corporation, Doha, Qatar

**Keywords:** Neisseria meningitidis, case report, septic arthritis, constrictive pericarditis, serogroup W

## Abstract

Invasive meningococcal disease is a life-threatening infection that classically presents as meningitis or meningococcemia. Extra-meningeal manifestations, including pericarditis and septic arthritis, are uncommon and may complicate diagnosis, particularly in the absence of meningeal involvement. A previously healthy vaccinated man in his 50s presented with acute bilateral knee pain and swelling following a short history of fever, productive cough, fatigue, and dyspnea. Blood cultures grew *Neisseria meningitidis* serogroup W, whereas cerebrospinal fluid, synovial fluid, and pericardial fluid cultures remained negative. Cardiac magnetic resonance imaging demonstrated constrictive pericarditis with circumferential pericardial enhancement and pericardial effusion. The patient was successfully treated with intravenous ceftriaxone, colchicine, ibuprofen, pericardiocentesis, and bilateral knee washout, followed by oral ciprofloxacin. This case adds to the limited literature describing concurrent cardiac and musculoskeletal manifestations of invasive meningococcal disease without meningitis and shows the importance of early recognition, comprehensive investigation and multidisciplinary management of these uncommon presentations.

## Introduction

Disseminated meningococcal infection is a severe form of invasive disease caused by *Neisseria meningitidis*.^1,2^ Although meningitis and meningococcemia remain the classical clinical presentations, invasive disease may also manifest with extra-meningeal complications, including pneumonia, septic arthritis, and pericarditis.^1,3^ Among the twelve identified serogroups, six (A, B, C, W, X, and Y) account for most invasive disease worldwide. In recent years, hypervirulent serogroup W, particularly clonal complex 11 (cc11) lineages, has emerged in several countries and has been associated with severe and atypical extra-meningeal manifestations.^1,4–6^ Septic arthritis is an uncommon manifestation of meningococcal disease, reported in approximately 2% of invasive meningococcal disease cases in a recent population-based study,^7^ although a 34-year single-centre cohort reported a higher incidence of 7.5%, suggesting that its frequency varies across different study populations and clinical settings.^8^ Cardiac manifestations, although clinically uncommon, may underestimate the true frequency of meningococcal cardiac involvement, as historical autopsy studies identified myocardial involvement in up to 78% of fatal cases.^9^ Recognition of these atypical extra-meningeal manifestations is important, particularly in vaccinated individuals and in the absence of meningitis. We report a case of disseminated *N. meningitidis* serogroup W infection presenting with bilateral septic arthritis and constrictive pericarditis in a previously healthy vaccinated adult, supported by clinical and multimodality imaging findings. This case report has been prepared in accordance with the CARE (CAse REport) guidelines,^10^ and patient details have been de-identified.

## Case presentation

A previously healthy male in his early 50s presented to the emergency department of Aisha Bint Hamad Al Attiyah Hospital, Doha, Qatar, in early-2026 with a one-day history of acute-onset bilateral knee pain and swelling resulting in an inability to ambulate. This was preceded by a two-day history of productive cough, fever, fatigue, and shortness of breath. He denied headache, neck stiffness, rash, chest pain, palpitations, nausea, or vomiting. His immunizations were up to date, including a documented single dose of the quadrivalent meningococcal conjugate vaccine (MenACWY-TT; Nimenrix®) administered 8 months before presentation, as part of occupational health requirements. He had no significant past medical or family history. He had no recent travel or known sick contacts and resided in shared accommodation. Written informed consent for treatment was obtained from the patient in accordance with institutional policy.

**Figure 1.**
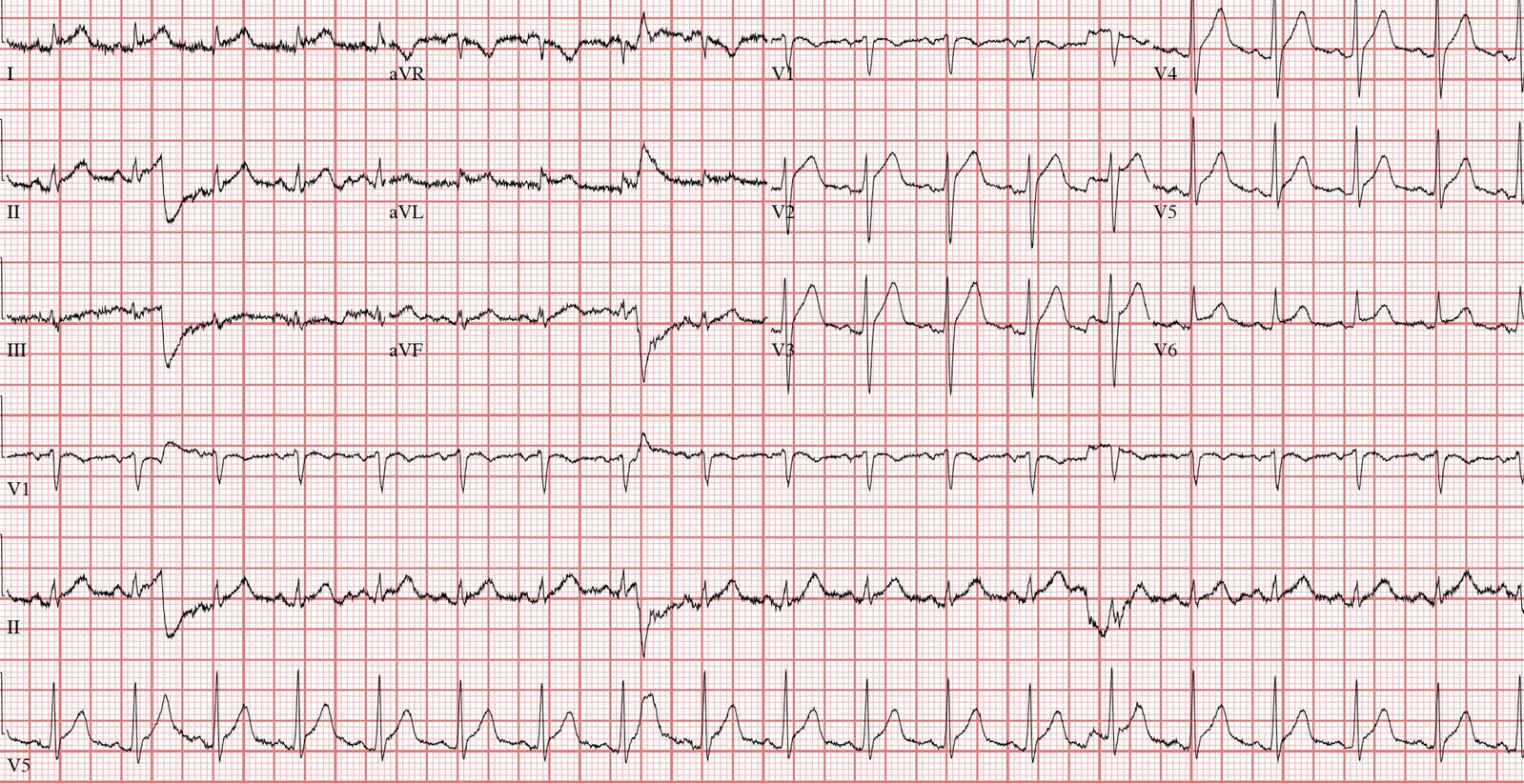
12-lead ECG demonstrating diffuse concave ST-segment elevation with PR depression consistent with acute pericarditis.

On admission, he was febrile (38.2°C) and tachycardic (112 beats/min). Examination revealed bilateral knee swelling with reduced range of motion, more pronounced on the right, and a pericardial friction rub, with no meningeal signs. Laboratory investigations (Table 1) demonstrated leukocytosis, thrombocytosis, markedly elevated C-reactive protein, elevated high-sensitivity troponin-T (48 ng/L), mild transaminitis, and mildly elevated procalcitonin (0.14 ng/mL). Autoimmune screening was negative, and serum immunoglobulin levels were within normal limits, terminal complement pathway screening (CH50) was not performed. Electrocardiography (ECG) demonstrated diffuse concave ST-segment elevation with PR-segment changes consistent with acute pericarditis (Figure 1), while chest radiography showed borderline cardiomegaly without focal pulmonary consolidation.

**Table 1.** Blood investigations.

Parameter	Value	Reference range
White blood cell count	13.5 ×10^3^/µL	4.0–11.0
Haemoglobin	12.4 g/dL	13.0–17.0
Platelets	461 ×10^3^/µL	150–400
C-reactive protein	322 mg/L	<5
Troponin-T high sensitivity	48 ng/L	<14
ALT	116 U/L	<40
AST	75 U/L	<40
Sodium	132 mmol/L	135–145
Procalcitonin	0.14 ng/mL	<0.05

Abbreviations: ALT, alanine aminotransferase; AST, aspartate aminotransferase.

Two sets of blood cultures were obtained from separate peripheral sites, one from each arm, and both were positive for *N. meningitidis* serogroup W. The findings were reported to public health authorities. Molecular typing (including clonal complex analysis) beyond serogroup determination was not performed. Despite the absence of meningeal signs and following recommendation of the infection disease team, a lumbar puncture was performed. Cerebrospinal fluid (CSF) analysis, including culture and viral PCR, showed no evidence of central nervous system infection. Bilateral knee ultrasound demonstrated joint effusions, and synovial fluid analysis revealed a neutrophil-predominant inflammatory effusion consistent with septic arthritis. Pericardial fluid was grossly bloody with neutrophil-predominant leukocytes. Gram stain and cultures were negative for both synovial and pericardial fluid. Respiratory viral PCR was positive for rhinovirus (Table 2).

**Table 2.** Microbiological and body fluid investigations.

Investigation	Findings
Respiratory viral PCR	Positive for human rhinovirus
Blood cultures	*N. meningitidis* serogroup W-135 isolated
Synovial fluid appearance	Turbid, light yellow
Synovial fluid white cell count	62,125 cells/µL (R) and 49,000 cells/µL (L)
Synovial fluid neutrophils	85%
Crystal analysis	Negative
Synovial fluid Gram stain/culture	Negative
Pericardial fluid appearance	Bloody
Pericardial fluid white cell count	11,400 cells/µL
Pericardial fluid neutrophils	76%
Pericardial fluid protein	4.0 g/dL
Pericardial fluid LDH	734 U/L
Pericardial fluid Gram stain/culture	Negative
CSF culture and viral PCR	Negative
Pericardial fluid culture	Negative

Abbreviations: CSF, cerebrospinal fluid; L, left knee; LDH, lactate dehydrogenase; PCR, polymerase chain reaction; R, right knee.

**Figure 2.**
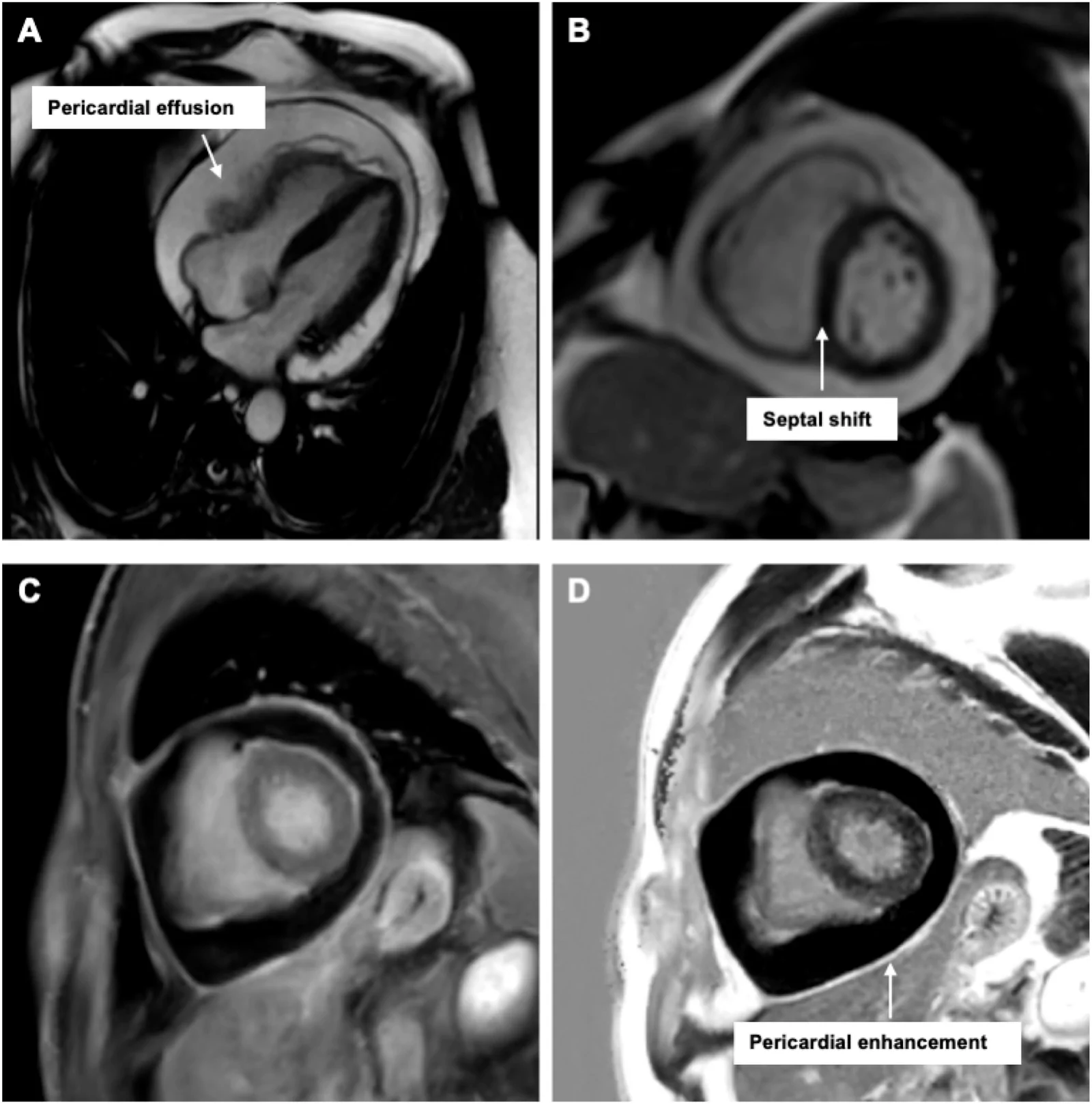
Cardiac magnetic resonance imaging demonstrating constrictive pericarditis. (A) Four-chamber cine image showing circumferential pericardial effusion. (B) Short-axis cine image demonstrating septal shift consistent with constrictive physiology. (C) T2-weighted short-axis image showing no myocardial edema. (D) Late gadolinium enhancement short-axis image demonstrating circumferential pericardial enhancement without myocardial enhancement.

Transthoracic echocardiography (TTE) demonstrated a moderate-to-large circumferential pericardial effusion with absence of valvular vegetations. Subsequent cardiac magnetic resonance (CMR) imaging demonstrated circumferential pericardial enhancement, moderate pericardial effusion, and interventricular septal shift consistent with constrictive physiology with preserved biventricular systolic function and no overt myocardial edema or myocardial late gadolinium enhancement (Figure 2). These findings were consistent with a clinical course of constrictive pericarditis.^11^ Viral pericarditis, autoimmune inflammatory disease, infective endocarditis, and myocarditis were initially considered; however, positive blood cultures for *N. meningitidis* serogroup W, neutrophil-predominant synovial fluid, negative autoimmune screening, absence of valvular vegetations, and CMR findings supported the clinical diagnosis of disseminated meningococcal infection with constrictive pericarditis and bilateral septic arthritis. Although troponin elevation raised concern for myocardial involvement, the absence of myocardial oedema and late gadolinium enhancement on CMR did not fulfill the updated Lake Louise criteria for definite myocarditis.^12^

**Figure 3.**
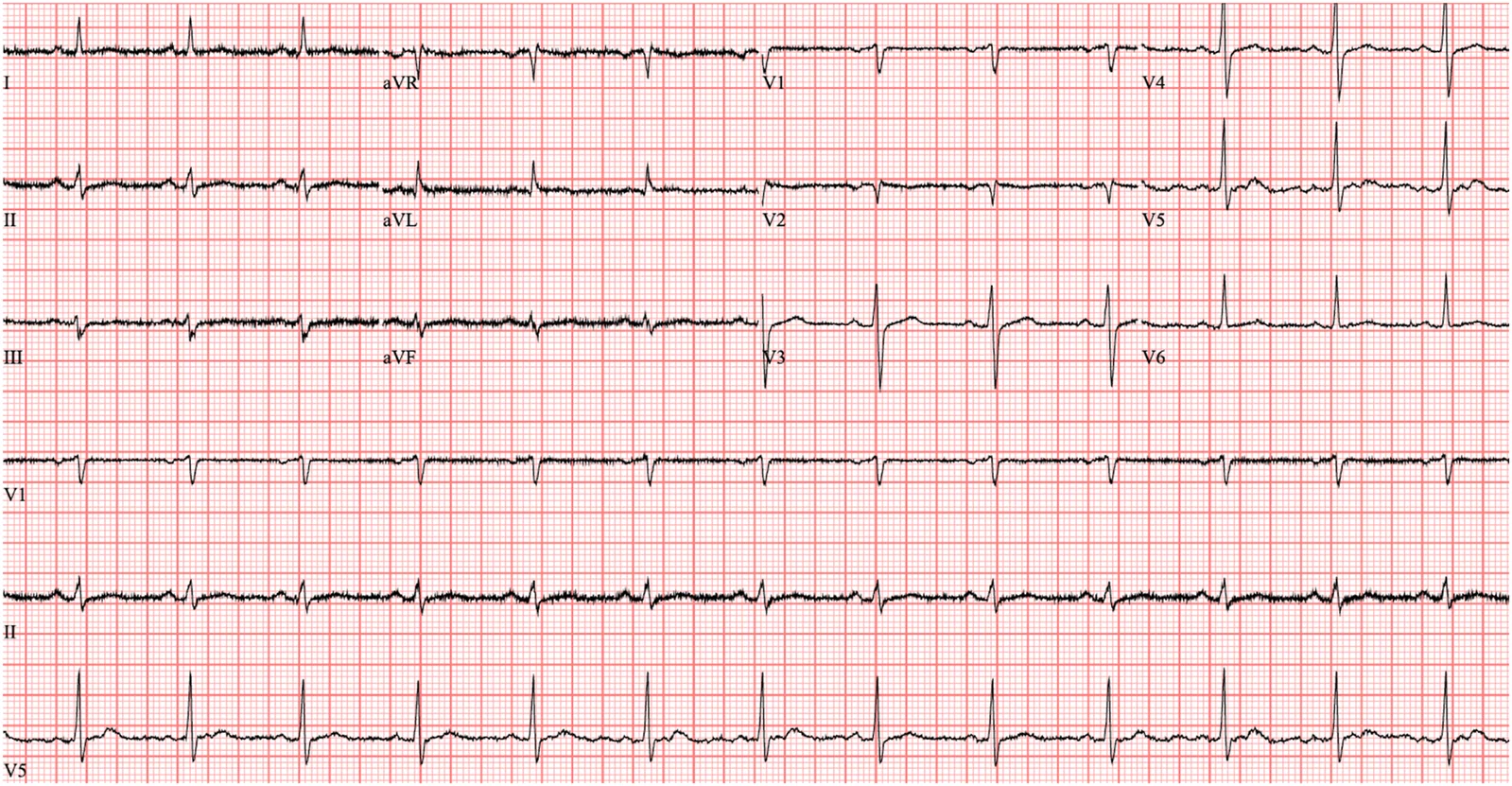
Follow-up 12-lead ECG demonstrating resolution of diffuse concave ST-segment elevation with PR depression following treatment.

The patient was commenced on ceftriaxone (2 g intravenously every 24 hours), colchicine (0.5 mg orally twice daily), and ibuprofen (600 mg orally three times daily), with close monitoring in the coronary care unit. Despite treatment, serial TTE demonstrated progression of the pericardial effusion with constrictive physiology, prompting pericardiocentesis. Persistent bilateral knee pain and swelling also required bilateral knee washout for source control. Pericardial and synovial fluid cultures remained negative despite confirmed meningococcemia. Following these interventions, the patient’s condition improved, with declining inflammatory markers and normalization of cardiac biomarkers. Ceftriaxone was continued for three weeks, followed by ciprofloxacin (500 mg orally twice daily) for a further three weeks based on isolate susceptibility, completing a six-week antimicrobial course. He was discharged after three weeks. At four-week orthopedic follow-up, he had returned to work with full knee range of motion, mild residual swelling, and no recurrence of musculoskeletal symptoms. Repeat ECG at six weeks demonstrated resolution of the initial abnormalities (Figure 3). However, repeat TTE or CMR imaging to confirm resolution of the constrictive physiology was not performed.

## Discussion

Extra-meningeal involvement is an uncommon but well-recognized manifestation of invasive meningococcal disease and may include articular and cardiac complications such as septic arthritis, pericarditis, and myocarditis.^13,14^ Two principal mechanisms have been proposed: direct hematogenous seeding of the synovium causing septic arthritis, and a delayed immune-mediated inflammatory process characterized by sterile synovial effusions developing after systemic infection or antimicrobial therapy.^13,14^ Immune complex deposition may also explain the concurrent occurrence of meningococcal arthritis and pericardial inflammation.

Respiratory viral PCR was positive for rhinovirus. Rhinovirus has been shown to facilitate bacterial invasion by disrupting airway epithelial barrier function.^15^ However, no clinical association has been demonstrated between rhinovirus and invasive meningococcal disease, and the available evidence is limited to pediatric populations.^16^ As *N. meningitidis* can independently traverse the respiratory epithelium,^17^ the contribution of rhinovirus in our adult patient remains uncertain.

Invasive meningococcal disease may involve the heart through a similar mechanism to septic arthritis. Cardiac involvement has been classified into disseminated infectious pericarditis, primary meningococcal pericarditis, and reactive pericarditis developing after systemic infection.^18,19^ Immune complex deposition may contribute to delayed serosal and myocardial inflammation, particularly in patients with concurrent arthritis and pericardial disease.^13^ In our patient, imaging findings and TTE evidence of constrictive physiology were consistent with constrictive pericarditis. The favourable clinical response to anti-inflammatory therapy was suggestive of a transient inflammatory constrictive phenotype; however, as follow-up TTE or CMR was not performed, resolution of the constrictive physiology could not be objectively confirmed. The coexistence of bilateral septic arthritis and cardiac involvement in our patient therefore represents an unusual extra-meningeal presentation of disseminated serogroup W disease.

Even with positive blood cultures confirming meningococcal infection, cultures from the other affected sites remained negative. Molecular testing for *N. meningitides* was not performed on synovial fluid, such testing may have improved microbiological confirmation, particularly after initiation of antimicrobial therapy.^20^ Therefore, although the temporal association with confirmed meningococcaemia supported meningococcal-associated articular and pericardial involvement, direct infection of these sites could not be distinguished from immune-mediated inflammation, and a contribution from the concurrent rhinovirus infection cannot be excluded. Management therefore required treatment of both the invasive infection and its inflammatory complications. Although uncomplicated meningococcemia is typically treated with a shorter duration of intravenous (IV) ceftriaxone (7 days),^21^ our patient received three weeks of IV ceftriaxone because of disseminated invasive disease complicated by bilateral septic arthritis. Current guidelines recommend shorter antibiotic courses for uncomplicated meningococcal septic arthritis. However, our patient had meningococcemia with bilateral septic arthritis and concomitant constrictive pericarditis. The six-week antimicrobial course was individualized by the treating infectious disease team based on the extent of disease and institutional practice. This approach is consistent with the conventional regimen described by Coakley et al., although the optimal treatment duration remains uncertain.^22^ While Gjika et al. demonstrated that two weeks of therapy was non-inferior to four weeks following surgical drainage of native joint septic arthritis, meningococcal arthritis was excluded, limiting extrapolation of these findings to our patient.^23^ After clinical improvement, treatment was transitioned to oral ciprofloxacin for an additional 3 weeks because the blood culture isolate demonstrated ciprofloxacin susceptibility, along with ciprofloxacin’s high oral bioavailability and favorable joint tissue penetration.^24^ Emerging evidence further supports early transition to targeted oral antimicrobial therapy after adequate surgical drainage in native joint septic arthritis.^23^

Although no previously published report exactly mirrors our presentation, Steele et al. described concurrent polyarticular septic arthritis and myopericarditis,^25^ whereas our patient developed constrictive pericarditis with CMR-confirmed constrictive physiology requiring pericardiocentesis, representing a distinct pattern of cardiac involvement. Previous reports have described cardiac involvement as pericarditis, myopericarditis, or myocarditis,^25–28^ and primary meningococcal septic arthritis as monoarticular or polyarticular disease managed with joint drainage and IV third-generation cephalosporins.^29–31^ Positive synovial fluid cultures have been reported in several arthritis cases, whereas cardiac involvement was more commonly confirmed by positive blood cultures with echocardiographic or CMR findings, and microbiological confirmation from pericardial fluid was uncommon.^25–28^ Most reported cases responded to high-dose IV ceftriaxone, although severe or progressive pericardial disease occasionally required drainage procedures and broader antimicrobial coverage.^25,32^ Similarly, our patient had positive blood cultures but sterile synovial and pericardial fluid cultures, with progression of constrictive physiology requiring pericardiocentesis and bilateral knee washout. Unlike previously reported myocarditis fulfilling the Lake Louise criteria,^27^ no myocardial oedema or late gadolinium enhancement was identified on CMR. Despite confirmed meningococcaemia, CSF cultures remained negative and no meningeal manifestations developed, possibly because of early ceftriaxone administration.

Despite prior vaccination, our patient developed invasive meningococcal serogroup W disease, an increasingly recognized cause of severe, atypical, and extra-meningeal presentations.^33^ Shared accommodation and prolonged close-contact exposure likely increased transmission risk, particularly given the high prevalence of asymptomatic nasopharyngeal carriage among healthy individuals.^34,35^ Although MenACWY conjugate vaccination programs have substantially reduced the incidence of vaccine-covered serogroups, breakthrough invasive disease may still occur despite prior immunization.^34^ The occurrence of serogroup W disease only eight months after MenACWY vaccination also raises the possibility of impaired complement-mediated protection. Terminal complement deficiencies involving C5–C9 markedly increase susceptibility to invasive meningococcal disease, and breakthrough infections may occur despite vaccination because effective meningococcal killing depends on an intact membrane attack complex.^33,36^ C7 deficiency associated with meningococcal disease has also been reported in Qatar.^37^ Complement testing was not performed in our patient; therefore, an underlying terminal complement deficiency cannot be excluded.

## Conclusion

Concurrent constrictive pericarditis and bilateral septic arthritis without meningitis represent an uncommon manifestation of invasive meningococcal disease that poses important diagnostic and therapeutic challenges. Although our patient had a favorable clinical outcome, several limitations should be acknowledged, including the single-case design, short follow-up period, the absence of molecular typing, lack of PCR testing on synovial and pericardial fluid, no CH50 testing, and the absence of follow-up CMR to confirm resolution of the constrictive physiology. In addition, negative site cultures prevented definitive distinction between direct bacterial infection and immune-mediated inflammation. We hope that acknowledging these limitations will encourage more comprehensive investigation of similar cases, allowing future reports to address these unanswered questions and improve our understanding of these uncommon manifestations.
